# Short-term ambient temperature increases are associated with higher mortality in amyotrophic lateral sclerosis: a case-crossover study of weather conditions

**DOI:** 10.3389/fnagi.2026.1868482

**Published:** 2026-08-06

**Authors:** Maximilian Dimitrov, Julian Grosskreutz, Benjamin Ilse, Florian Rakers, Robert Steinbach

**Affiliations:** 1 Department of Neurology, Jena University Hospital, Jena, Germany; 2 Precision Neurology of Neuromuscular and Motor Neuron Diseases, University of Luebeck, Luebeck, Germany; 3 Cluster for Precision Medicine in Inflammation, Universities of Kiel and Luebeck, Luebeck, Germany; 4 MVZ Neurologie, MVZ Zentralklinik Bad Berka, Weimar, Germany

**Keywords:** amyotrophic lateral sclerosis, case-crossover study, mortality risk, temperature rise, weather conditions, thermoregulation, heat related mortality, end of life care

## Abstract

**Introduction:**

Amyotrophic lateral sclerosis (ALS) is a neurodegenerative disorder with vastly differing survival times. The influence of environmental exposures such as weather conditions on mortality among people living with ALS remain poorly defined. The aim of this study was to evaluate the association between short-term meteorological phenomena and the odds of death among patients with ALS.

**Methods:**

We conducted a retrospective case-crossover study of 298 deaths among clinically verified ALS patients that occurred within a weather-station catchment area. Daily mean data for temperature, atmospheric pressure, and relative humidity were analyzed. Associations were analyzed by comparing hazard intervals (1–3 days before death) with bidirectional control intervals in generalized linear mixed models. Results are expressed as odds ratios (OR) and 95% confidence intervals (CI).

**Results:**

Absolute meteorological conditions (temperature, pressure, humidity) showed no significant association with mortality, which was independently supported by seasonal/monthly death count analyses. Short-term temperature rises of 3 °C in the 24 h preceding the day before death were associated with 25% higher odds of death (OR 1.25; 95% CI 1.05–1.49). Exploratory subgroup analyses suggested that this association was most pronounced in female, ventilated, and older patients (≥65 years). Changes in relative humidity and atmospheric pressure were not significantly associated with the odds of death.

**Conclusion:**

Short-term temperature increases are associated with higher odds of death among people living with ALS, independent of absolute weather conditions or seasonal patterns. These findings support attention of stable ambient temperatures in end-stage care, while further studies of weather-related hazards in ALS are needed.

## 1 Introduction

Amyotrophic lateral sclerosis (ALS) is a neurodegenerative disease characterized by marked heterogeneity in progression patterns and disease duration. Survival averages approximately 2–5 years after symptom onset but can vary substantially across patients (Westeneng et al., 2018). Many factors associated with prognosis of people living with ALS (pALS) have been identified and modifiable factors are increasingly addressed in multidisciplinary care (Vasta et al., 2025; Traynor et al., 2003). Nevertheless, survival gains at the population level remain inconsistent across cohorts, suggesting that additional clinical or environmental hazards may contribute to mortality in advanced disease (McFarlane et al., 2023; Vasta et al., 2025; Forbes et al., 2004).

This observation indicates that current interventions may be insufficient to prolong life expectancy for all pALS at cohort level and may also reflect unmeasured or increasingly relevant external factors. Identifying such hazards is important for improving therapeutic management but also to appraise the usefulness of survival as an outcome in interventional and observational ALS studies.

Autopsy and clinical cause-of-death studies suggest that respiratory failure is the most frequently reported terminal pathway in ALS, whereas aspiration pneumonia is reported less consistently (Corcia et al., 2008; Kurian et al., 2009; Gil et al., 2008; Spataro et al., 2010). However, exact terminal mechanisms are often incompletely adjudicated, and comorbid acute events may contribute to individual deaths. Seasonal increase in mortality has been suggested, as, e.g., related to respiratory infections during winter. However, Pinto and De Carvalho (2017) found no overall relative increase in monthly death counts in a retrospective cohort of 543 pALS.

To our knowledge, no previous study has linked real meteorological data to mortality among pALS. Accordingly, this study assessed absolute meteorological conditions and short-term weather changes in association with the odds of death in the course of ALS. If hazardous or protective weather patterns can be identified, the findings may inform anticipatory counselling, home-care planning, and environmental precautions in end-stage ALS care.

## 2 Materials and methods

### 2.1 Study design and cohort

This investigation was designed as a case-crossover study. We retrospectively identified patients from a tertiary care center specialized in the treatment of pALS, located at Jena University Hospital in the state of Thuringia, Germany. The study region is characterized by a temperate climate. Electronic datasets of 523 deceased adult patients with neuromuscular diseases (aged ≥ 18 years) who died between May 2009 and July 2023 were extracted from a local specialized database (Steinbach et al., 2020). All patients signed written informed consent and the procedures were approved by the local Ethics Committee of the Friedrich Schiller University in Jena (3633–11/12). Subjects’ consent was obtained according to the Declaration of Helsinki and its later amendments. Each patient’s medical file was individually reviewed to verify the ALS diagnosis according to the Gold Coast criteria (Shefner et al., 2020). Cases with clear competing terminal conditions such as life-shortening malignancy or suicide were excluded. The precise date and postal code of the location of death were obtained from reports by relatives or healthcare professionals to the center in Jena or corroborated through publicly available death announcements. Cases were excluded if date or location of death was missing or uncertain, if the diagnosis was not ALS after file review, if death occurred more than 40 km from the nearest meteorological station, or if ALSFRS-R data required for clinical disease-course evaluation were missing.

Additional information was extracted from the database: sex, age (at the day of death), region of first symptom onset (bulbar, limb, thoracic or generalized), survival time (defined as months from symptom onset until death), status of ventilation (was any invasive or non-invasive ventilation initiated or not?), status of feeding tube (implanted or not), and D50 as a patient-specific time-constant depicting overall disease aggressiveness (Steinbach et al., 2021; Meyer et al., 2025). Meteorological data (24 h daily averages) for the entire study period were obtained from the German Meteorological Service’s (DWD) publicly accessible Climate Data Center. We utilized data from 24 meteorological monitoring stations where deaths among clinically verified ALS patients occurred within a 40 km radius around the weather station. These measurements were treated as standardized ambient exposure proxies.

The investigation was reported according to the STROBE criteria.

### 2.2 Statistical methods

To determine whether short-term weather phenomena were associated with the odds of death among pALS, we employed a bidirectional case-crossover analysis. It was specifically developed to assess acute effects of short and transient exposure (Navidi, 1998; Bender and Lange, 2001). In this model, statistical inference is based on within-subject comparisons, where meteorological conditions during a “hazard interval” immediately preceding the event of interest (here: death) are compared with conditions during “control intervals.” The hazard interval was defined as the −1 to −3-day-period immediately preceding the patient’s death. Bidirectional control intervals were defined as the same corresponding weekdays in the week prior to and the week following death to account for potentially confounding time-trends in exposure conditions in this case-only study. This model assesses whether the odds of death are higher when weather conditions in the hazard period systematically differ from those in the control period. Generalized Linear Mixed Models (GLMM) were applied under two distinct scenarios. First, the impact of prevailing weather was studied, by analyzing mean daily values of meteorological parameters one day before death, 2 days before death, and 3 days before death (lag-times of 1, 2 or 3 days respectively). Second, the impact of rapid weather changes was analyzed by assessing 24-h changes between 2 days and one day before death (lag-time of 1 day), between 3 days and 2 days before death (lag-time of 2 days), and between 4 days and 3 days before death (lag-time of 3 days). The time intervals were selected to account for a potential delay before ambient weather conditions affect the odds of death (Rakers et al., 2017).

This self-matching approach inherently controls for time-invariant confounders as each participant acts as their own control, thereby eliminating all stable personal characteristics, such as chronic comorbidities, socioeconomic status, or baseline functional impairment (Rakers et al., 2017; Kockler et al., 2021). This approach mirrors the logic of conditional logistic modeling traditionally used in such designs but is implemented here through a mixed-effects structure to accommodate clustering by weather station. Bidirectional selection of control days was applied to counteract gradual seasonal drifts, long-term meteorological cycles, or weekly behavioral patterns that could otherwise bias exposure comparisons. Matching on the same weekday additionally mitigates day-of-week effects in environmental conditions and healthcare-seeking behavior.

The association between meteorological exposures and mortality among pALS was quantified using Odds Ratios (OR) and corresponding 95% Confidence Intervals (CI). Ambient temperature, atmospheric pressure, and relative humidity were treated as fixed effects. The individual patient and meteorological station were treated as random effects. Effect estimates were expressed per interquartile-range (IQR) change in each meteorological variable, ensuring comparability of effect sizes and aligning the interpretation with established practice in environmental epidemiology. Model stability and adequacy were evaluated by inspecting random-effect variance components and checking for irregularities in residual patterns, analogous to diagnostics recommended for self-matched environmental analyses.

All statistical calculations were performed in the R environment (version 4.4.3 for Windows 11 platform), using the *glmer* function from the *lme4* 1.1–36 package. To minimize the risk of bias from multiple testing or selective reporting, all meteorological variables, hazard and control intervals, outcome definitions, and statistical tests were pre-specified before the analysis was conducted. Data were first analyzed for the entire study cohort. To minimize the risk of Type I error due to multiple testing across the six primary models (three lag-times for both absolute values and day-to-day changes), a conservative Bonferroni-adjusted alpha level of 0.0083 was considered for the total population. Secondary exploratory subgroup analyses were conducted for pre-specified categories based on: sex (male versus female), age at death (<65 years versus ≥65 years), D50 depicting overall disease aggressiveness (high aggressiveness with D50 < 30 versus low aggressiveness with D50 ≥ 30) (Steinbach et al., 2021; Meyer et al., 2025), ventilated (yes versus no), and survival time (separated by cohort-specific median: ≤30 months versus >30 months).

Furthermore, we conducted chi-square analyses to evaluate whether the distribution of deaths varied significantly across calendar months or seasons as previously described (Pinto and De Carvalho, 2017). Observed frequencies were compared against the expected counts derived under the assumption of a uniform distribution. For monthly comparisons, the expected distribution corresponded to 25 deaths per month, whereas for seasonal analyses, the expected distribution corresponded to 75 deaths per season.

Non-normal distribution of continuous variables was examined using the Shapiro-Wilks test. Skewed variables are reported as median with inter-quartile range, categorical variables are given as absolute numbers and percentages. Comparison of group-wise averages was appropriately conducted either with a Mann–Whitney U-test or a chi-square test.

## 3 Results

### 3.1 Study population

**Figure 1:**
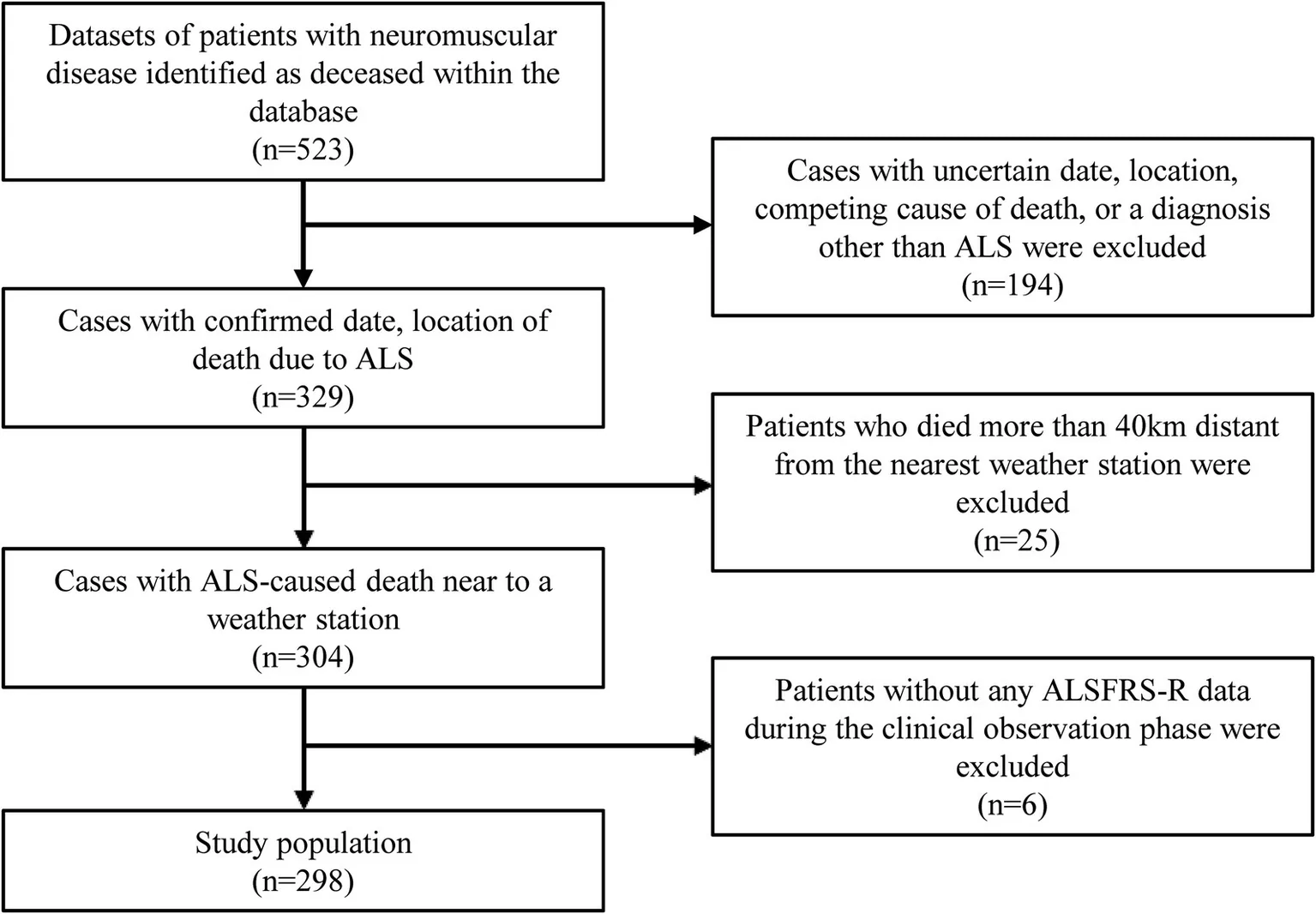
Flowchart of patient selection for the final study cohort. From the 523 patients documented as deceased, 194 cases were excluded due to missing or uncertain information regarding date or location of death, a diagnosis other than ALS, or clear competing terminal conditions such as life-shortening malignancy or suicide. Subsequently, 25 cases were excluded because death occurred more than 40 km from the nearest weather station. Another 6 patients were excluded because ALSFRS-R data were missing, preventing evaluation of clinical disease course and D50. The final study cohort consisted of 298 pALS whose characteristics are summarized in Table 1.

A summary of the demographic and clinical characteristics of the final study population is given in Table 1. The distributions of sex, age, region of onset, and survival time were typical for pALS treated in European tertiary centers (Westeneng et al., 2018; Steinbach et al., 2020; Grassano et al., 2024). In comparison with the 225 excluded cases, the included cohort did not differ significantly regarding sex, age, D50, gastrostomy status, survival time, or region of first symptom onset (Supplementary Table S1). However, a slightly smaller fraction of the excluded cases had a documented initiation of ventilatory support (43.56% vs. 56.38% in the included cohort; *p* = 0.0049). This difference should be interpreted with caution, because the excluded cases were primarily those with incomplete follow-up data, and thus carried greater uncertainty concerning the exact date and place of death Figure 1.

**Table 1:** Summary of study population of 298 patients with ALS.

Median [IQR] (range)		*n*	%
All patients		298	100
Sex			
Male		171	57.38
Female		127	42.62
Age [years]	68.29 [60.1–74.73] (36.75–86.75)		
≥ 65 years		180	60.4
< 65 years		118	39.6
D50 value	23.72 [15.54–32.76] (0.28–333.04)		
D50 < 30 months		207	69.46
D50 ≥ 30 months		91	30.54
Ventilated patients			
Yes		168	56.38
With Tracheostomy		12	4.03
No		129	43.29
Patients with gastrostomy		124	41.61
Survival time [months]	30 [21–45] (4–372)		
≤ 30 months		156	52.35
> 30 months		142	47.65
Region of onset			
Bulbar		110	36.91
Limb		182	61.07
Thoracic		4	1.34
Generalized		2	0.67

Categorial variables are given as numbers (*n*) and percentages. Continuous variables are averaged as median, the interquartile ranges [IQR] and total ranges (minimum**—**maximum) are given in brackets.

### 3.2 Meteorological variables

A summary of meteorological variable distributions collected during the observation period from all contributing weather stations is given in Supplementary Table S2. In summary, the weather patterns recorded in the study catchment were characteristic of a temperate climate, featuring mild summer conditions and relatively cool winters.

### 3.3 Scenario 1: mortality odds in association with absolute weather conditions

**Figure 2:**
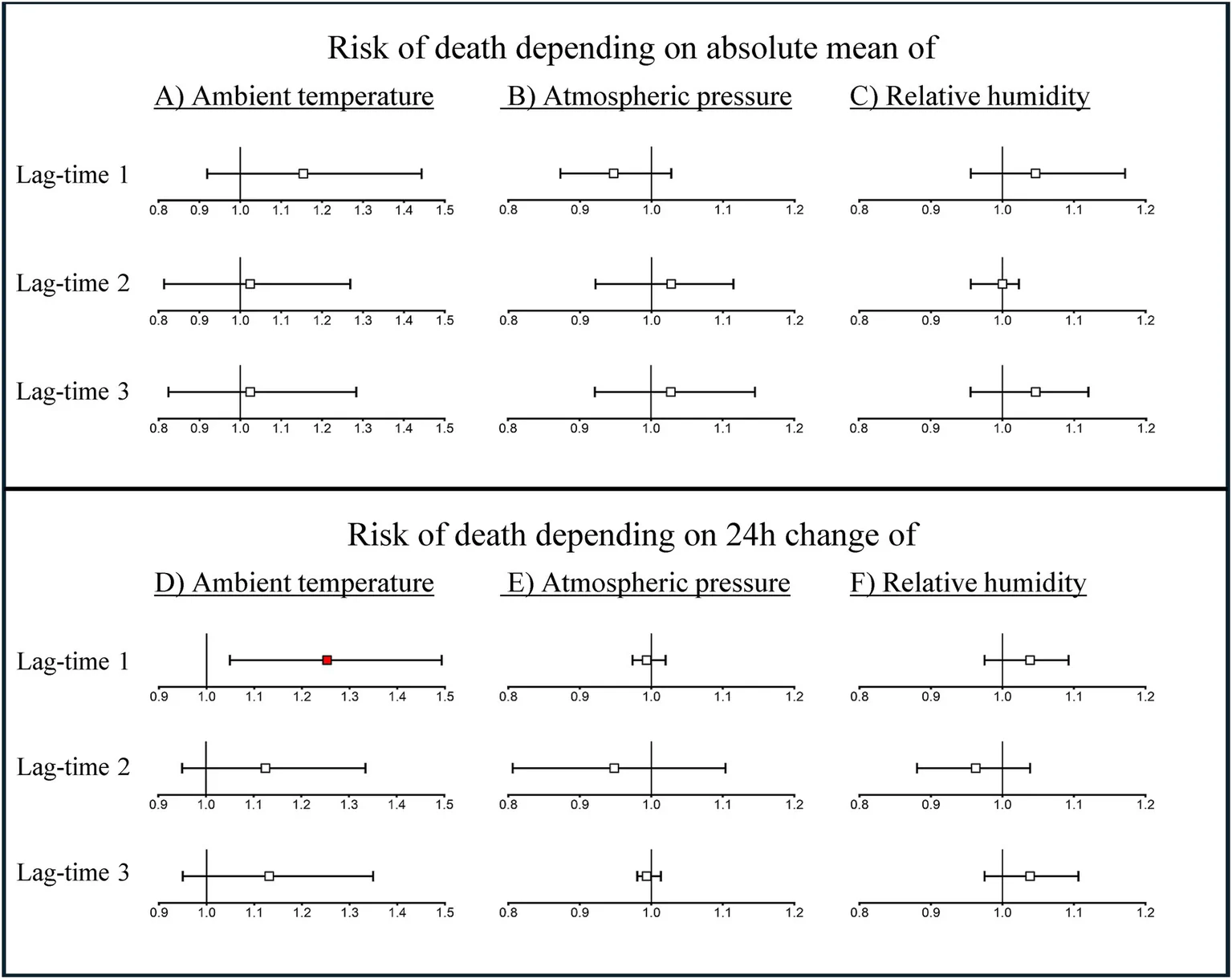
Odds of death in relation to absolute daily meteorological parameters and in relation to 24-h meteorological changes for all patients. Forest plots depict odds ratios (OR) and whiskers mark 95% confidence intervals (CI) for associations between the absolute daily values of **(A)** temperature, **(B)** atmospheric pressure, **(C)** relative humidity (right panels) and the odds of death. No significant associations were revealed. The OR represent the change in odds associated with an interquartile range increase in ambient temperature of 12.03 °C, relative air humidity of 22.67%, and atmospheric pressure of 27.18 hPa. Analyses are presented for the day before death (lag time of 1 day; top row), 2 days before death (lag time of 2 days; middle row), and 3 days before death (lag time of 3 days; bottom row). Associations between 24-h changes in **(D)** temperature, **(E)** atmospheric pressure, **(F)** relative humidity and the odds of death. An association was observed for temperature-change at lag-time 1 (marked in red). The OR represent the change in odds associated with an interquartile range 24-h change in ambient temperature of 3 °C, relative air humidity of 12.67%, and atmospheric pressure of 6.6 hPa. Analyses refer to the 24-h change between 2 days and one day before death (lag time of 1 day; top row), the change between 3 days and 2 days before death (lag time of 2 days; middle row), and the change between 4 days and 3 days before death (lag time of 3 days; bottom row).

Analyzing potential linear effects, we found no significant association between any meteorological parameter (temperature, pressure, or humidity) and the odds of death among patients with ALS (pALS) at any lag time (Figures 2A–C). All odds ratios were close to 1 and displayed no consistent pattern in either direction.

### 3.4 Scenario 2: mortality odds in association with 24 h changes in weather conditions

**Figure 3:**
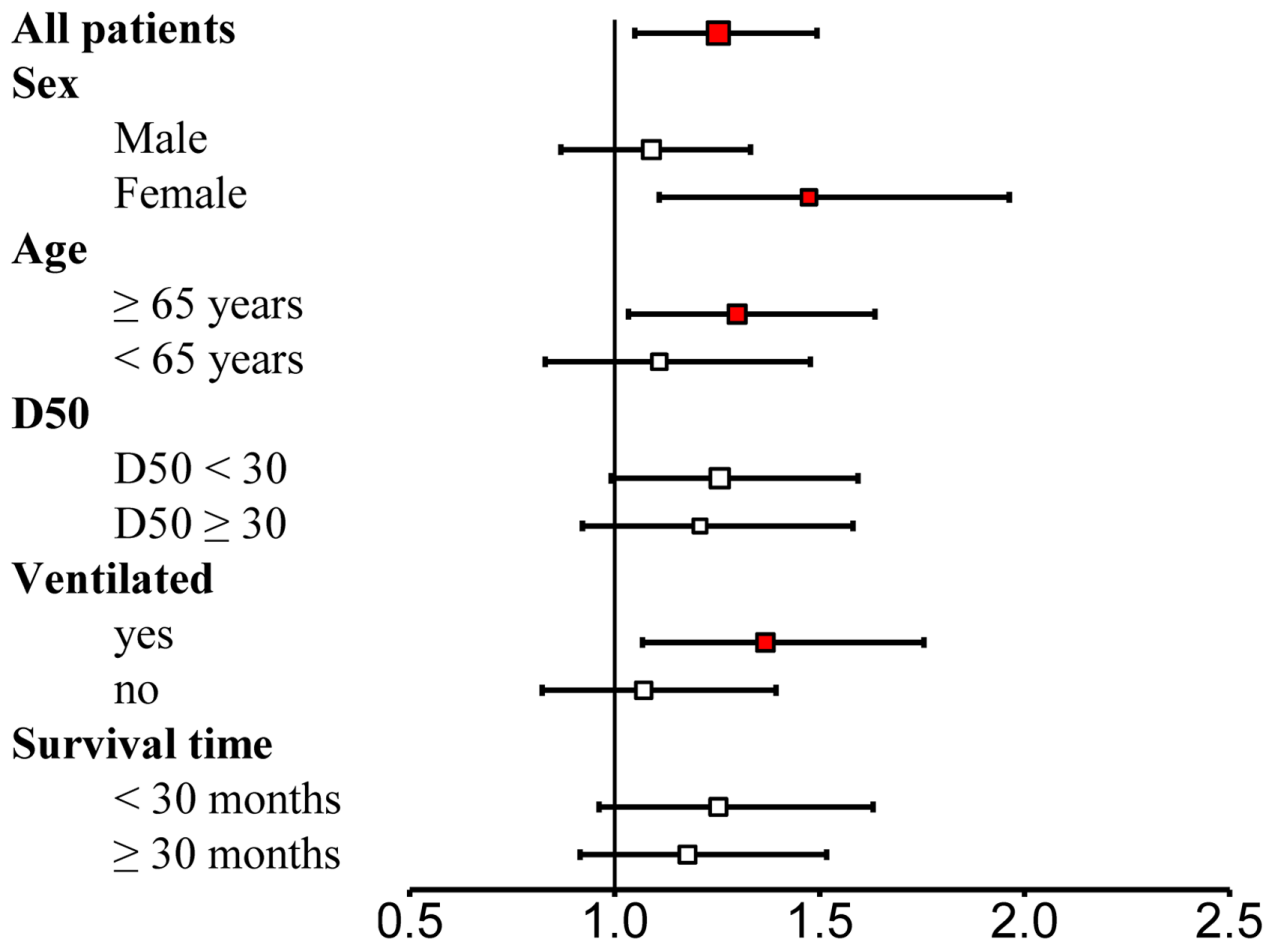
Subgroup analyses for the odds of death in relation to 24-h changes of temperature at lag-time 1. Forest plots depict odds ratios (OR) and whiskers mark 95% confidence intervals (CI) for associations with the 24-h temperature change between 2 days and one day before death (lag time of 1 day). Associations were observed for the subgroups of female, ventilated, and older patients with ALS (aged ≥ 65 years) at the time of death (marked in red). The OR represent the change in odds associated with an interquartile range 24-h change in ambient temperature of 3 °C.

In the overall study cohort of 298 pALS, a 24-h increase in ambient temperature of 3 °C from day −2 to the day preceding death (lag time of one day) was associated with 25% higher odds of death (OR 1.25; 95% CI 1.05–1.49) (Figure 2D). A similar association of rising temperatures in the 24 h preceding the day before death was observed in exploratory subgroup analyses of female, ventilated, and older pALS aged ≥ 65 years (Figure 3). Among females, the odds of death were 47% higher (OR 1.47; 95% CI 1.11–1.96), while ventilated patients had 37% higher odds (OR 1.37; 95% CI 1.07–1.75), and patients aged ≥65 years had 30% higher odds (OR 1.30; 95% CI 1.03–1.64).

No other significant associations were identified. In particular, changes in relative humidity over 24 h showed odds ratios close to 1, with no consistent directional trend at any lag time.

### 3.5 Analysis of death counts in relation to time during the year

Comparing monthly and seasonal death counts to a uniform distribution, no significant deviations were found, indicating that mortality did not vary systematically by month or season. Monthly death counts ranged from 19 to 30 deaths per month (chi-square test, *p* = 0.943). Seasonal death counts were similarly balanced (winter 77, spring 69, summer 78, autumn 74; *p* = 0.883; Supplementary Table S3).

## 4 Discussion

In this study, we analyzed the potential hazardous or protective influences of short-term meteorological exposures up to 3 days before death on mortality risk in patients with ALS (pALS). Daily mean absolute values of ambient temperature, relative humidity, and atmospheric pressure were not associated with the odds of death for pALS (Figures 2A–C), indicating that mortality is independent of the general weather period. In line with this, a complementary analysis comparing monthly and seasonal death counts did not reveal any seasonal clustering during the year (Supplementary Table S3). In contrast, day-to-day temperature increases were associated with higher odds of death, especially for the 24 h preceding the day of death (i.e., day −2 up to day −1; Figure 2D). This association, observed in the overall cohort, persisted in exploratory subgroups of older, ventilated, and female patients (Figure 3).

ALS is a relentlessly progressive disease, characterized by highly variable inter-individual survival times. Respiratory failure remains the most frequently reported terminal pathway in small autopsy series (Corcia et al., 2008; Kurian et al., 2009). Nevertheless, detailed peri and post-mortem studies remain raw. Consequently, investigating environmental conditions preceding death is of particular interest to expand the knowledge and improve end-stage care for pALS. Interestingly, prior reports of long-term survivors undergoing home tracheotomy mechanical ventilation indicated a higher prevalence of sudden unexpected deaths (Sancho et al., 2011). Acute events such as dehydration, infection, or sudden cardiac arrest, may contribute to individual deaths. In addition, co-morbidities are often underrecognized in advanced ALS, owing for instance to limited access to specialists or atypical/hidden symptom presentations, such as exertional dyspnea (Pinto et al., 2012; Finsterer et al., 2016). The relevance for end-stage care remains high because advanced ALS is characterized by reduced respiratory reserve, limited mobility, impaired cough and swallowing, nutritional vulnerability, and dependence on caregivers or ventilation—all of which compromise the ability to compensate for acute thermal stress (Dupuis et al.,2018; Finsterer et al., 2016; Riku et al., 2021).

This study provides strong evidence that within the studied cohort of pALS, the odds of death were independent of absolute meteorological conditions (Figures 2A–C). This is further corroborated by the complementary analysis of monthly and seasonal death counts, which indicates that deaths occurred evenly throughout the year (Supplementary Table S3). At first glance, this finding appears counterintuitive, as pALS are exceptionally susceptible to respiratory infections that naturally occur more frequently during winter in the studied region. However, protective measures might mitigate exposure to infection in the end-stages of ALS. Notably, the case-crossover design applied in this study addresses confounding by long-term temporal trends, as hazard intervals are compared with nearby bidirectional control intervals for the same individual. Nevertheless, acute infections, changes in care access, and epidemic or pandemic shifts in the virulence of respiratory pathogens remain plausible residual contributors to individual deaths. Regarding the latter, the observation period overlapped with the COVID-19 pandemic, although the existing literature on COVID-19-related mortality in ALS and neuromuscular diseases supports a nuanced interpretation. While patients with respiratory impairment are clinically more vulnerable and pandemic-associated restrictions disrupted multidisciplinary care, published ALS cohorts have reported heterogeneous effects on mortality (Andrews et al., 2020; Costamagna et al., 2021; Garcia-Casanova et al., 2024; Raymond et al., 2025; Capozzo et al., 2020; Sole et al., 2020; Tseng and Chen, 2021). In our center, adapted triage implemented at the onset of COVID-19 restrictions helped preserve advanced care planning, including the timely initiation of non-invasive ventilation and gastrostomy. This proactive management may have contributed to maintaining stable standards of care in the present cohort during the pandemic period (Steinbach et al., 2020). As a further exploratory overview, a comprehensive summary of death counts cross-referenced by month and year alongside aggregated meteorological data did not suggest any temporal alignment with either COVID-19 waves or months characterized by weather conditions that typically promote viral dissemination (Supplementary Table S4).

The main observation of this study remains the association between short-term temperature rises and higher odds of death (Figure 2D). Biologically, this finding might be partly attributable to an interplay with the onset of infections in pALS, as these individuals may experience a steeper fever-associated rise in core body temperature and subsequent rapid decompensation. However, this hypothesis requires further investigation. Beyond the biological mechanisms, there are practical implications for end-stage ALS care. Potential measures include counseling patients and caregivers about the impact of rapid temperature increases, avoiding poorly insulated rooms, implementing shading and ventilation strategies, ensuring hydration where clinically appropriate, and considering cooling capacity when planning home care, hospice care, or transfer to long-term care facilities. Notably, the present analysis examined death as a distinct outcome because the case-crossover design requires precise event dates. It remains plausible that rapid temperature increases may also contribute to non-fatal motoneuron disease-associated morbidity, such as the duration of ventilator dependence or hospital admissions. However, evaluating these outcomes will require other study designs, such as prospective multicenter studies.

The subgroup analyses (Figure 3) suggested that female patients may be particularly vulnerable to rising temperatures, with 47% higher odds of death (OR 1.47; 95% CI 1.11–1.96) for every 3 °C increase in temperature in the 24 h before the day of death. This aligns with physiological studies indicating sex-based differences in thermoregulation, which demonstrate that women often exhibit a lower sweat rate and less efficient heat dissipation (Leach et al., 2024). It is well known that ALS pathology affects the hypothalamus early in the course of the disease, where the brain’s thermoregulatory center is located (Dupuis et al., 2018; Vernikouskaya et al., 2023). This dysfunction might well exacerbate the inherent challenges female pALS face in heat management, thereby leading to a higher vulnerability to short-term temperature rises.

Increasing temperatures were also more strongly associated with mortality among patients receiving ventilatory support, who exhibited 37% higher odds of death (OR 1.37; 95% CI 1.07–1.75 for every 3 °C increase in ambient temperature). Most ventilated patients in this study were treated with non-invasive ventilation, either provided via mask or high-flow nasal cannula, which provides less consistent conditioning of inspired air than invasive ventilation. As a result, higher ambient temperatures may exacerbate airway dryness, discomfort, sleep disruption, and alterations in gas conditioning. Studies on respiratory support indicate that temperature and humidification can potentially influence patient comfort and patient-ventilator interaction, although ALS-specific data remain limited (Mauri et al., 2018; Bongiovanni et al., 2021).

The increased susceptibility to thermal stress among patients aged ≥ 65 years (30% higher odds of death; OR 1.30; 95% CI 1.03–1.64) aligns with broader European evidence indicating that older adults are disproportionately affected by heat (Ballester et al., 2023; Winklmayr et al., 2022). These findings are particularly relevant, given that ALS incidence rises with age and the ongoing demographic shift toward an ageing population is expected to increase the number of individuals living with the disease (Arthur et al., 2016).

Altogether, the results also highlight the potential implications of climate warming for the morbidity and overall mortality among pALS. A nationwide case-crossover study from China investigated temperature-related mortality burdens across neurodegenerative diseases and projected a substantial rise in heat-related deaths under high-emission scenarios (Yin et al., 2023). Furthermore, empirical observations from heatwave studies indicate that heat-related deaths typically occur within narrow lag windows (0 to 3 days), especially among older populations (Winklmayr et al., 2022; Liu et al., 2024).

We also observed a non-significant trend toward lower odds of death with decreasing atmospheric pressure (Supplementary Figure S1). Although this observation should be interpreted cautiously, it may reflect the interconnected nature of weather conditions, as patients were simultaneously exposed to all three meteorological variables. Consequently, the observed effect might reflect the same underlying phenomenon, given that falling atmospheric pressure likely prevents short-term temperature rises (due to adiabatic cooling). However, since this finding was not statistically significant, it should be regarded as exploratory.

The main strength of this study is the bidirectional case-crossover design, which evaluates meteorological conditions immediately preceding death (hazard interval) while using each patient as their own control by means of an intra-individual comparison with weather patterns from the surrounding time (control interval). This self-matching effectively controls for time-invariant patient characteristics such as sex, baseline age, socioeconomic context, chronic comorbidity burden, long-term medication, and baseline functional impairment—over the short observation interval (Mittleman and Mostofsky, 2014). Additional strengths include clinically verified ALS diagnoses, rigorous individual validation of death dates and locations, standardized weather data from official monitoring stations (DWD), the assessment of both absolute meteorological values and day-to-day changes, independent analyses of monthly/seasonal death counts, and a long observation period conducted at a specialized ALS center.

Several limitations should be considered. First, the model relies on ambient meteorological data from central monitoring stations and therefore cannot account for individual microclimate exposure. This study lacks systematic data on indoor temperatures, insulation, heating or cooling behaviors, air conditioning, patient mobility, and time spent outdoors. Therefore, these weather-station measurements should be interpreted as standardized ambient exposure proxies rather than direct measurements of each patient’s immediate thermal environment. However, this limitation does not affect atmospheric pressure, as it remains uniform indoors and outdoors. Moreover, extensive heating or air conditioning is uncommon in the examined region, and patient mobility was likely highly restricted, given that the majority of patients with end-stage ALS are completely housebound or bedridden.

Second, the use of 24-h daily averages for meteorological variables limits the exposure resolution. Consequently, we cannot evaluate extreme weather conditions that peak within a single day, nor can we assess the distinct impacts of compound heatwaves or tropical nights, although some of these phenomena may be partially reflected in daily averages. While such effects have previously been associated with population-based, heat-related spikes in mortality, conflicting results exist in the literature, which may be attributable to a “harvesting effect” (Winklmayr et al., 2022; Frasch et al., 2025). Such accelerated short-term mortality among highly vulnerable groups likely also applies to patients in advanced stages of ALS, but this hypothesis requires further investigation using a larger—ideally multicenter—dataset.

Third, there might be non-linear associations between meteorological conditions and the odds of death in pALS. Exploratory analyses applying previously described methodologies did not reveal any significant effects (data not shown), but such potential dynamics warrant further investigation using larger datasets or multicenter cohorts (Rakers et al., 2017; Kockler et al., 2021; Gasparrini et al., 2010).

Fourth, while a detailed medical file review was performed for all included cases, we cannot entirely rule out that underrecognized or undiagnosed comorbid conditions (such as cancer) contributed to the cause of death (Sato et al., 2007; Riku et al., 2021).

Finally, the excluded cases showed a slightly smaller proportion of patients with a documented ventilation status, which is likely attributable to restricted follow-up data in this group. Still, a potential selection bias cannot be fully ruled out, particularly regarding the exploratory subgroup analyses stratified by ventilation status (Riku et al., 2021).

Altogether, our study demonstrates that short-term temperature rises are associated with higher odds of death among pALS. This finding has important implications for patients’ advance care planning, suggesting that setting-specific precautions to maintain stable temperatures should be considered toward the end-stage of the disease. Independent, ideally multi-center studies are required to determine whether this potential impact of rising temperatures can be replicated. However, this would require careful methodological planning, as the case-crossover approach applied here strictly requires certainty about exact death dates and locations. Direct indoor-exposure measurements, e.g., through wearables, would provide valuable resources but require carefully planned prospective future studies. Moreover, regional differences in climate, cultural influences on care, and patient-centric decisions about the end of life should be considered in future work (Andersen et al., 2018; Barc et al., 2022).

## Data Availability

The raw data supporting the conclusions of this article will be made available by the authors, without undue reservation.
