# Supplementary material for: Short-term ambient temperature increases are associated with higher mortality in amyotrophic lateral sclerosis: a case-crossover study of weather conditions
Source: Front Aging Neurosci. 2026 Aug 6;18:1868482. doi: 10.3389/fnagi.2026.1868482 (PMC13490180; doi:10.3389/fnagi.2026.1868482)
Supplement: Supplementary file 1 [file Data_Sheet_1.docx]

Supplementary Material

# 1 Supplementary Table 1

| Comparison of included patients (n = 298) and excluded cases (n = 225). | | | | |
| --- | --- | --- | --- | --- |
| **Subgroups** | **Included cohort** | **Excluded cases** | **Test** | **p-value** |
| Male sex | 171/298 (57.38%) | 121/225 (53.78%) | Chi-square | 0.4636 |
| Female sex | 127/298 (42.62%) | 104/225 (46.22%) | — | — |
| Age ≥65 years | 180/298 (60.40%) | 149/220 (67.73%) | Mann–Whitney U | 0.5059 |
| Age <65 years | 118/298 (39.60%) | 71/220 (32.27%) | — | — |
| D50 <30 months | 207/298 (69.46%) | 121/201 (60.20%) | Mann–Whitney U | 0.0676 |
| D50 ≥30 months | 91/298 (30.54%) | 80/201 (39.80%) | — | — |
| Ventilated patients | 168/298 (56.38%) | 98/225 (43.56%) | Chi-square | 0.0049* |
| Patients with gastrostomy | 124/298 (41.61%) | 88/225 (39.11%) | Chi-square | 0.6266 |
| Survival time <30 months | 156/298 (52.35%) | 108/215 (50.23%) | Mann–Whitney U | 0.7376 |
| Survival time ≥30 months | 142/298 (47.65%) | 107/215 (49.77%) | — | — |
| **Region of onset** | | | | |
| Bulbar | 110/298 (36.91%) | 67/212 (31.60%) | Fisher's exact | 0.2214 |
| Limb | 182/298 (61.07%) | 136/212 (64.15%) | Fisher's exact | 0.5167 |
| Thoracic | 4/298 (1.34%) | 5/212 (2.36%) | Fisher's exact | 0.4999 |
| Generalized | 2/298 (0.67%) | 4/212 (1.89%) | Fisher's exact | 0.2392 |
| *Overall distribution* | 298 | 212 | Fisher–Freeman–Halton | 0.3180 |

**Table 1. Comparison of the subgroups of the included cohort (n = 298) and the excluded cases (n = 225).** Values are given as absolute counts with the corresponding percentage within each cohort. Sex, ventilation and gastrostomy-status were compared with the chi-square test; age, D50 and survival time were compared with the Mann–Whitney U test. Region of onset was compared with Fisher’s exact test for each individual subgroup (that subgroup vs. all others) and with the Fisher–Freeman–Halton exact test for the overall 2 × 4 distribution. The denominator of the excluded cases is not always 225, because data were missing for some patients in the individual subgroups and for the region of onset; each variable was therefore analyzed in the number of excluded patients for whom the respective information was available (age: n = 220; D50: n = 201; survival time: n = 215; region of onset: n = 212).

# 2  Supplementary Table 2

| **Summary of Meteorological Variable Distributions from May 16, 2009, to August 15, 2023.** | | | | | |
| --- | --- | --- | --- | --- | --- |
|  | **Quartiles** | | | | |
| **Meteorological variables** | min. | 1^st^ | median | 3^rd^ | max. |
| Temperature (°C) | -16.4 | 3.5 | 9.1 | 15.5 | 29.0 |
| Atmospheric Pressure (hPa) | 888.9 | 959.5 | 975.3 | 986.3 | 1020.7 |
| Relative Humidity (%) | 28.3 | 64.6 | 77.2 | 87.0 | 100 |

# 3 Supplementary Table 3

| **Monthly and Seasonal analyses of death counts for the study cohort of 298 pALS.** | | |
| --- | --- | --- |
| **month** | **deaths per month** | **deaths per season** |
| Dec | 23 | 77 (winter) |
| Jan | 29 |  |
| Feb | 25 |  |
| Mar | 23 | 69 (spring) |
| Apr | 21 |  |
| May | 25 |  |
| Jun | 27 | 78 (summer) |
| Jul | 23 |  |
| Aug | 28 |  |
| Sep | 19 | 74 (autumn) |
| Oct | 30 |  |
| Nov | 25 |  |
| ***p-value*** | 0.943 | 0.883 |

# 4 Supplementary Table 4

Exploratory overview of death counts per month and year together with monthly avergaed weather data aggregated across the 24 regional DWD monitoring stations. The descriptive look did not suggest that winter-peaking respiratory pathogens drove deaths in this cohort: During the colder and drier winter months (which typically favor viral dissemination), deaths remained sparse, whereas months with multiple events often exhibited milder temperatures (above 0°C). The COVID-19 infection waves did also not suggest coincidence with death-rates. However this has to be intpreted catiously, because 1) Aggregating meteorological factors across an entire month mathematically obliterates critical short-term dynamics, such as acute day-to-day temperature spikes. 2) As a monocentric cohort spanning from May 2009 to July 2023, the sample size per individual month is naturally too small to permit any robust statistical evaluation.

# Supplementary Figure 1

# 5 Supplementary Figure 2

.
